# Chemical data of contaminants in water and sediments from the Doce River four years after the mining dam collapse disaster

**DOI:** 10.1016/j.dib.2022.108715

**Published:** 2022-11-09

**Authors:** F.Y. Yamamoto, G.E. Pauly, L.S. Nascimento, G.M. Fernandes, M.P. Santos, B.S.M. Kim, M.U. Carvalho, R.C.L. Figueira, R.M. Cavalcante, M.T. Grassi, D.M.S. Abessa

**Affiliations:** a Institute of Biosciences, São Paulo State University, São Vicente, Brazil; b Marine Science Institute, Federal University of Ceará, Fortaleza, Brazil; c Chemistry Department, Federal University of Paraná, Curitiba, Brazil; d Oceanographic Institute, University of São Paulo, São Paulo, Brazil

**Keywords:** Metals, PAHs, Brazil, Contamination, Iron tailing

## Abstract

Chemical datasets describing the occurrence of both inorganic and organic contaminants along the Doce River Basin (DRB) could provide a better understanding of the potential impacts of a major mining dam collapse disaster combined to additional chronic sources of contamination. This data article presents datasets of main contaminants detected in the water and sediments sampled four years after the mining dam collapse in the DRB. A summary table of data obtained in the literature is also provided to allow a comparison of the variation of chemicals before, right after in 2015/2016 and after the event (current data). In addition, there are also provided physical-chemical parameters of water and sediments of different sampling sites, which could support the investigation of chemicals distribution. For this purpose, triplicate samples of water and sediment were obtained in 8 sampling sites along the DRB during wet and dry seasons of 2019, totalizing 48 samples of each environmental matrix. The sampling sites were strategically selected according to their different main sources of pollution along the river. Concentrations of trace elements and organic contaminants (polycyclic aromatic hydrocarbons, and pyrethroids) were determined in samples of water and sediments by inductively coupled plasma mass spectrometry (ICP-MS) and gas chromatography - mass spectrometry GC-MS, respectively. Main data obtained in the literature consisted in published reports from environmental agencies (IGAM) and private companies (RENOVA) as well as journal articles. The datasets provided may be useful to the stakeholders, which include scientific community, authorities and public agencies, and private companies interested to understand the impacts of the contaminants introduced along the River Basin four years after the environmental disaster.

**Specifications Table**

**Table:** 

Subject	Environmental Sciences - Pollution
Specific subject area	Environmental contaminants - Detection and quantification of inorganic and organic contaminants in field samples of water and sediment by analytical chemistry.
Type of data	Tables
How the data were acquired	Physical-chemical parameters of water Water – Data available in the literature from RENOVA (https://www.fundacaorenova.org/arquivos-e-relatorios) and IGAM websites (http://www.igam.mg.gov.br/monitoramento-da-qualidade-das-aguas2/monitoramento-da-qualidade-das-aguas-superficiais-do-rio-doce-no-estado-de-minas-gerais). Sediment properties Sediment – Data generated from sediment samples obtained from the Doce River (MG/ES, Brazil). The analysis of composition and grain size distribution of sediment was performed following the wet and dry sieving method. The quantity of Organic Matter (OM) was determined through the method of wet organic matter oxidation. Chemical analyses Inorganic chemicals Data were acquired by an inductively coupled plasma mass spectrometry (ICP-MS) Organic chemicals Data were acquired by GC-MS and HPLC-DAD
Data format	Raw Analyzed
Description of data collection	Data was generated from the analysis of samples of water and sediment obtained in two field surveys of the Doce River, located in Southeast Brazil, during wet and dry seasons of 2019. In each survey, eight sampling sites were established across the DRB, and three replicates of water and sediment samples were collected, in both wet and dry seasons.
Data source location	*For Tables 3-9 in Chemical data folder and the Raw data folder:* 1. *Institution:* Institute of Biosciences, São Paulo State University 2. *City/Town/Region:* São Vicente, São Paulo 3. *Country: Brazil* 4. *Latitude and longitude (and GPS coordinates, if possible) for collected samples/data:*
	1. Gualaxo do Norte (Mariana, MG) – 20°16′44.9″S 43°25′50.2″W 2. Candonga (Rio Doce, MG) - 20°12′24.1″S 42°52′39.7″W 3. Naque (Naque, MG) - 19°14′37.5″S 42°19′09.1″W 4. Rio Corrente (Periquito, MG) - 19°01′31.0″S 42°09′37.3″W 5. Governador Valadares (Governador Valadares, MG) - 18°51′22.0″S 41°55′49.5″W 6. Aimorés (Aimorés, ES) - 19°30′25.0″S 41°00′53.5″W 7. Colatina (Colatina, ES) - 19°30′51.8″S 40°44′21.7″W 8. Linhares (Linhares,ES) - 19°24′30.2″S 40°02′47.4″W The data of physical-chemical parameters of water were obtained in the literature from: six weekly reports for the wet season and three monthly reports for the dry season published by RENOVA (Table 1, Chemical data folder); and for IGAM (Table 2, Chemical data folder) data, it was used four reports from wet and four for the dry seasons of 2019. The reported data of inorganics concentrations detected in water and sediments by different previous studies, were searched in the PubMed database, and their references are described in the tables S1 and S2 (Review of literature data folder).
Data accessibility	Repository name: Mendeley Data [1] Chemical datasets of environmental samples (2019) from the Doce River Basin, Brazil Data identification number: 10.17632/sf2h83t8v3.4 Direct URL to data: https://data.mendeley.com/datasets/sf2h83t8v3/4
Related research article	*Published in Science of the total Environment* *-Yamamoto, F.Y^.^, Costa Souza, A.T., Paula, V.C.S., Beverari, I., Garcia, J.R.Q., Padial, A.A., Abessa, D.M.S, 2022.* From molecular endpoints to modeling longer-term effects in fish embryos exposed to the elutriate from Doce River., Sci. Tot. Env. https://doi.org/10.1016/j.scitotenv.2022.157332

## Value of the Data

• Relevant datasets are provided for screening assessment of major inorganic and organic contaminants occurring along the Doce River basin, and their distribution, three to four years after the mining dam collapse disaster
• Data exploration may be useful to distinguish the impacts from multiple sources of pollutants present along the river that have been affecting the river basin before and after the event, and thus better clarifying the distribution of these contaminants four years after the event.
• The datasets are relevant for performing a more appropriate environmental diagnostic to better predict further ecological and human risks of exposure, including the cumulative effects due to the disaster and the contribution of other pollution sources.
• The data will be useful for the assessment of public authorities (environmental agencies), riverine communities, private companies affected by the mining dam disaster, as well as scientific community, which are concerned by the impacts of the mining dam rupture and are still investigating such impacts.
• The data can be further explored by the application of environmental diagnostic indexes and multiple statistical tools to evidence the major contaminants of concern, the most impacted regions and identify the main sources of pollution (causes) that are affecting the local water quality.
• Chemical data can be associated and integrated with biological data to understand potential impacts of these chemicals in exposed organisms [2]. Finally, these data can be used in comparisons focusing the contamination levels before and after the dam rupture.

## 1 Data Description

**Raw data** folder:

• **Grain size Sediment _ UNESP.xlsx** (excel table): Includes initial total weights of each sample, as well as the weight after the sieving process. The tables include the calculation to obtain the percentages of each class of grain size (Clay and silt, very fine sand, fine sand, medium sand, coarse sand, very coarse sand)
• **Inorganics Filtered Water raw data - USP.pdf**: Includes the values of the metal(oids) obtained from the ICP instrument, including standard curve calibrations, blancks and the 48 samples of water of the Doce River (Solutions concentrations, units, Standard Deviation and %RSD).
• **Inorganics filtered water_USP.xls** (excel table): Concentrations of metal(loid)s (Al, As Ca, Cd, Cr, Cu, Fe, K, Mn, Ni, Pb, Sc, Sr, Ti, V, Zn) and their respective Relative Standard Deviation (%RSD) determined by ICP, in 48 water samples (mg.L^−1^) of the Doce River Basin. Maximum, minimum, median and average values for each variable considering all samples are shown in the bottom of the table.
• **Inorganics in Sediments - USP.xls (excel table)**: Concentrations of metal(loid)s (Al, As, Cd, Cr, Cu, Fe, Hg, Mn, Ni, Pb, Sc, Ti, V, Zn) and their respective Relative Standard Deviation (%RSD) determined by ICP, in 48 samples of sediments (mg.L^−1^) of the Doce River Basin. Maximum, minimum, median and average values for each variable considering all samples are shown in the bottom of the table.
• **Inorganics sediment raw data - USP.pdf**: Includes the values of the metal(oids obtained from the ICP instrument, including standard curve calibrations, blancks and the 48 samples of sediments of the Doce River (Solutions concentrations, units, Standard Deviation and %RSD).
• **LQ-LD-PAHs - UFC.xlsx** (excel table): Limits of quantification (LQ) and Limits of Detection (LD) for the PAHs measured in water and sediments samples of the Doce River.
• **PAHs sediments.zip**: Folder containing raw data of the chromatograms obtained by the GC-MS for the analysis of PAHs in all sediment samples
• **PAHs Sediments - UFC.xlsx** (excel table): Tables containing peak areas of sediment samples and internal standards, used for the calculation of the concentrations of PAHs in the samples.
• **PAHs water GC-MS.zip**: Folder containing raw data of the chromatograms obtained by the GC-MS for the analysis of PAHs in all filtered water samples
• **PAHs Water- UFC.xlsx** (excel table): Tables containing peak areas of water samples and internal standards, used for the calculation of the concentrations of PAHs in the samples.
• **Pyrethroids - UFC.xlsx** (excel table): Tables containing peak areas of water and sediment samples and internal standards, used for calculation of concentrations of Pyrethroids in the samples.
• **Pyrethroid.zip**: Folder containing raw data of the chromatograms obtained by GC-MS for the analysis of Pyrethroids in filtered water and sediment samples
• **Repeating analisys of Al and Fe after dilution - USP.xls**: Table of repetition of the analysis of Al and Fe after a second dilution factor, since the first measurement exceeded values of the standard curve.
• **Repeating analisys of Al and Fe after dilution.pdf**: Includes the values of the Al and Fe obtained from the ICP instrument, including standard curve calibrations, blancks and the 48 samples of water of the Doce River (Solutions concentrations, units, Standard Deviation and %RSD) after a second dilution factor.
• **Tables - Doce River 2019.docx**: tables containing all raw data in the word document.
• **Total inorganics in water - UFPR.xlsx**: Concentrations of metal(loid)s (Al, As, Cd, Co, Cu, Fe, Hg, K, Li, Mg, Mn, Na, Ni, Pb, Ti, V, Zn) and their respective Relative Standard Deviation (%RSD) determined by ICP, in 48 water samples (mg.L^−1^) of the Doce River Basin. Calculations of the concentrations and limits of quantifications are included in the excel file.

## 2 Chemical Data (Excel Tables 1-9) Folder

Table 1. Physical-chemical parameters of the water (Clorophyll a (µg.L^−1^), Cyanobacteria (µg.L^−1^), Conductivity (µS.cm^−1^) Temperature (°C) Turbidity (UNT), Dissolved O_2_ (mg.L^−1^) pH, Precipitation. (mm)) obtained from RENOVA, described for six different sites (GUA, CAN, NAQ, GOV, AIM, REG) along the DRB during wet and dry seasons of 2019.

Table 2. Physical-chemical parameters of water (dissolved Al, As, Cd, Pb, dissolved Cu, Cr, dissolved Fe, Mn, Hg, Ni, NO3, Zn, total Coliforms, Clorophyll a (µg.L^−1^), Cyanobacteria (µg.L^−1^), Conductivity (µS.cm^−1^) Temperature (°C) Turbidity (UNT), Dissolved O_2_ (mg.L^−1^) pH) obtained from IGAM, described for five different sites (GUA, CAN, NAQ, GOV, AIM) along the DRB during wet and dry seasons of 2019.

Table 3. Grain size distribution of sediment samples surveyed along the DRB during wet and dry seasons of 2019 determined by electromagnetic sieve shaker used to separate into 6 different sandy fractions, according to Wentworth (1922) classification. Organic Matter content of sediment samples, surveyed along the DRB during wet and dry seasons of 2019, determined through the method of wet organic matter oxidation according to [3], modified by [4].

Table 4. Concentrations of metal(loid)s (Al, As Ca, Cd, Cr, Cu, Fe, K, Mn, Ni, Pb, Sc, Sr, Ti, V, Zn) determined in acidified filtered water (mg.L^−1^), according to Procedure 1, sampled in eight locations along the DRB during wet and dry seasons of 2019.

Table 5. Concentrations of elements (Al, As, Cd, Co, Cu, Fe, Hg, K, Li, Mg, Mn, Na, Ni, Pb, Ti, V, Zn) determined in centrifuged water samples (mg.L^−1^), according to Procedure 2, obtained in eight locations along the DRB during wet and dry seasons of 2019.

Table 6. Concentrations of metal(loid)s (Al, As, Cd, Cr, Cu, Fe, Hg, Mn, Ni, Pb, Sc, Ti, V, Zn) determined in sediments (μg.g^−1^) sampled in eight locations along the DRB during wet and dry seasons of 2019.

Table 7. The Concentrations of the sixteen High Priority Polycyclcic Aromatic Hydrocarbons: Naphtalene (NAP), acenaphthylene (ACY), acenaphthene (Ace), fluorene (Flu), phenanthrene (Phe), anthracene (Ant), fluoranthene (Fltr), pyrene (Pyr), benzo[a]anthracene (BaA), chrysene (Chry), benzo[b]fluoranthene (BbF), benzo[k]fluoranthene (BkF), benzo[a]pyrene (BaP), benzo[g,h,i]perylene (BghiP), indeno[1,2,3-c,d]pyrene (Ind), and dibenz[a,h]anthracene (DahA) and other 5 non-priority PAH: 1-Methylnaphthalene (1-MNaf), 2-Methylnaphthalene (2-MNaf), Dibenzothiophene (DBT), benzo[e]pyrene (BeP), Perylene (Per) quantified in the water (μg.L^−1^) of the eight sampling sites along the DRB during the wet and dry seasons of 2019.

Table 8. The Concentrations of the sixteen High Priority Polycyclcic Aromatic Hydrocarbons: Naphtalene (NAP), acenaphthylene (ACY), acenaphthene (Ace), fluorene (Flu), phenanthrene (Phe), anthracene (Ant), fluoranthene (Fltr), pyrene (Pyr), benzo[a]anthracene (BaA), chrysene (Chry), benzo[b]fluoranthene (BbF), benzo[k]fluoranthene (BkF), benzo[a]pyrene (BaP), benzo[g,h,i]perylene (BghiP), indeno[1,2,3-c,d]pyrene (Ind), and dibenz[a,h]anthracene (DahA) and other 5 non-priority PAH: 1-Methylnaphthalene (1-MNaf), 2-Methylnaphthalene (2-MNaf), Dibenzothiophene (DBT), benzo[e]pyrene (BeP), Perylene (Per) detected in sediments (μg.kg^−1^) of the eight sampling sites along the the DRB during the wet and dry seasons of 2019.

Table 9. The Concentrations of Pyrethroids: Permetrhin (PER), Cyfluthrin (CYF), Cypermethrin (CYP), Deltamethrin (DEL), Bifenthrin (BIF) and summation of total Pyretrhoid (∑PYR) detected in the water (µg.L^−1^) and sediment (μg.kg^−1^) of the eight sampling sites along the the DRB during the wet and dry seasons of 2019.

## 3 Review of Literature Data

Table S1 – Water: summarizes the data obtained in the literature by Pubmed database, describing the levels of inorganic chemicals present in the water along different sites of the Doce River Basin before and after the dam rupture event in 2015.

Table S2 – Sediments: summarizes the data obtained in the literature by Pubmed database, describing the levels of inorganic chemicals present in sediments along different sites of the Doce River Basin before and after the dam rupture event in 2015.

Table 1. Physical-chemical parameters of the water obtained from RENOVA, described for six different sites along the DRB during wet and dry seasons of 2019.

Table 2. Physical-chemical parameters of water obtained from IGAM, described for five different sites along the DRB during wet and dry seasons of 2019.

Table 3. Granulometry and Organic Matter content of sediment samples surveyed along the DRB during wet and dry seasons of 2019.

Table 4. Concentrations of main inorganic chemicals detected in the water (in mg.L^−1^) of the DRB during wet and dry seasons of 2019, by Procedure 1.

Table 5. Concentrations of main inorganic chemicals detected in the water (in mg.L^−1^) of the DRB during wet and dry seasons of 2019, by Procedure 2.

Table 6. Concentrations of major trace elements detected in sediments (μg.g^−1^) of the DRB during the wet and dry seasons of 2019

Table 7. Concentrations of Polycyclcic Aromatic Hydrocarbons detected in the water (μg.L^−1^) of the DRB during the wet and dry seasons of 2019.

Table 8. Concentrations of Polycyclcic Aromatic Hydrocarbons detected in sediments (μg.kg^−1^) of the DRB during wet and dry seasons of 2019.

Table 9. The Concentrations of Pyrethroids detected in the water (µg.L^−1^) and sediment (μg.kg^−1^) of the eight sampling sites along the the DRB during the wet and dry seasons of 2019.

Table S1. Table summarizing the data obtained in the literature describing the levels of inorganic chemicals present in the water along the Doce River Basin before and after the dam rupture event in 2015.

Table S2. Table summarizing the data obtained in the literature describing the levels of inorganic chemicals present in sediments along the Doce River Basin before and after the dam rupture event in 2015.

## 4 Experimental Design, Materials and Methods

### 4.1 Materials and methods

The datasets contain concentrations of inorganic and organic contaminants determined in water and sediment samples obtained in 8 sampling sites along the Doce River, during wet and dry seasons of 2019. The eight sampling sites represent each of the three regions of the DRB (Yamamoto et al. 2022): Upper, Middle (both in the Minas Gerais state) and Lower Regions (Espírito Santo state), which are under the influence of different human activities [5].

Description of the sampling sites according to Yamamoto et al., (2022):

**Table:** 

Region	Sampling sites	Description of Human activities along the DRB [5]
Upper DR	1 - Gualaxo do Norte River(GUA), Mariana, MG	Intense mining activity, with iron and artisanal gold extraction. Closest site to the Fundão dam collapse, in Mariana municipality, MG.
	2- Candonga (CAN), HPP Reservoir, Rio Doce, MG	Hydropower Plant Reservoir that retained approximately 10 million m^3^ of mining tailing introduced into Doce River.

Middle DR	3 - Naque, MG (**NAQ**)	Downstream site of steel mill industries and a large-scale pulp mill industry (CENIBRA)
	4 - Reference site (**REF**), Corrente River, Periquito, MG	Doce River affluent selected as Reference site. The river cross a region within a restrictive Environmental Protected Area (Corrente River State Park), and was not affected by the mud wave.
	5 - Governador Valadares (**GOV**), MG	Largest urban center of Doce River basin (245 thousand hab.), with non-treated wastewater release
Low DR	6 - Aimorés, ES (**AIM**)	Crops (mainly coffee) and livestock
	7 - Colatina, ES (**COL**)	Crops and urban impact (Colatina district)
	8 - Linhares, ES (**LIN**)	Crops and urban impact (Linhares district)

The seasonal variation was also assessed in two sampling surveys performed during the wet season (Jan – Feb) and dry season (Jul-Aug) of 2019. The water samples (3 L per sampling site and season, divided in three field replicates of 1 L each) were directly collected with the own bottle for storage, at a depth approximately below 30 cm from the surface, in three different sites of each location. For the analysis of inorganic contaminants, the water samples (1 L per sample in triplicate) were acidified with HNO_3_ 0,5% within 24 h after sampling, and stored in new polypropylene bottles at 4° C, in the absence of light. For the organics, the water was stored in new amber glass bottles, and kept at 4° C in the absence of light. The surface sediment samples were obtained with plastic shovels, more than 5 meters distant from the riverbank and in a maximum depth of 10 cm, and stored in white polyethylene bottles for inorganic analysis, and in aluminum containers in the case of aliquots for organic compounds analyses, kept at -20° C until analysis. Samples of approximately 1 kg were collected in three different sites for each location along the Doce River (total of 3 kg per location, for triplicate analysis).

### 4.2 Physicochemical parameters of water

Data of physicochemical parameters were obtained by weekly and monthly reports available in the literature, from RENOVA Foundation and from the environmental agency IGAM, for the same periods of the field surveys of Wet (01/14/2019 to 01/23/2019 for sampling sites in MG state and 02/10/2019 to 02/18/2019 in ES state) and Dry (07/10/2019 to 07/19/2019 for sampling sites in MG state and 12/08/2019 to 18/08/2019 in ES state) of 2019. A total of six weekly reports for the wet season and three-monthly reports for the dry season published by RENOVA described Clorophyll a (mg/L), Cyanobacteria (mg/L), Conductivity (mS/cm), temperature (°C), turbidity (NTU), Dissolved oxygen (mg/L), pH and average precipitation (mm) in water. The search for physicochemical data considered six sampling sites described in the reports, which could correspond spatially to at least six out of the eight sites described in the field surveys. Regarding the IGAM data, four reports from Wet and four for the Dry described the levels of: (dissolved Al, As, Cd, Pb, dissolved Cu, Cr, dissolved Fe, Mn, Hg, Ni, NO_3_, Zn, total Coliforms, Clorophyll a (µg.L^−1^), Cyanobacteria (µg.L^−1^), Conductivity (µS.cm^−1^) Temperature (°C) Turbidity (UNT), Disolved O_2_ (mg.L^−1^) pH) at 5 different sites (GUA, CAN, NAQ, GOV, AIM) across the DRB. The quali-quantitative parameters of the water (QQPM) were obtained through an automatic monitoring of fixed equipments distributed along the Doce River Basin.

References for database for physicochemical parameters:

**Table:** 

Report		Repository link
**RENOVA Database**		
1	01/14/2019	https://www.fundacaorenova.org/wp-content/uploads/2019/01/boletim-semanal-periodo-chuvoso_14.01.2019.pdf
2	01/21/2019	https://www.fundacaorenova.org/wp-content/uploads/2019/01/boletim-semanal21.pdf
3	01/28/2019	https://www.fundacaorenova.org/wp-content/uploads/2019/01/boletim-semanal-periodo-chuvoso_28.01.2019.pdf
4	02/04/2019	https://www.fundacaorenova.org/wp-content/uploads/2019/02/boletim-semanal-periodo-chuvoso_04.02.2019.pdf
5	02/11/2019	https://www.fundacaorenova.org/wp-content/uploads/2019/02/boletim-semanal-periodo-chuvoso_11.02.19.pdf
6	02/18/2019	https://www.fundacaorenova.org/wp-content/uploads/2019/02/boletim-semanal-periodo-chuvoso_18.02.2019.pdf
7	02/25/2019	https://www.fundacaorenova.org/wp-content/uploads/2019/02/boletim-semanal-periodo-chuvoso_25.02.2019.pdf
8	07/03/2019	https://www.fundacaorenova.org/wp-content/uploads/2019/07/boletim-mensal-periodo-seco_junho_2019-1.pdf
9	08/06/2019	https://www.fundacaorenova.org/wp-content/uploads/2019/08/boletim-mensal-periodo-seco_julho_2019.pdf
10	09/09/2019	https://www.fundacaorenova.org/wp-content/uploads/2019/09/boletim-mensal-periodo-seco_agosto_2019.pdf
**IGAM database**		
1	2010-2019	http://www.igam.mg.gov.br/component/content/article/16-duvidas/2493–tabelas-de-resultados

### 4.3 Physicochemical parameters of the sediment samples

#### 4.3.1 Sediment grain size distribution

The composition and grain size distribution of the sediment was analyzed following the wet and dry sieving method (CETESB, L6.160). A fraction of the lyophilized sediment sample was weighed (100- 120 g per sample) and wet sieved (distilled water) in a 0.062 mm mesh to remove muddy fraction, being subsequently dried for 2 days at 60°C. Then, the material was sieved for 20 min in an electromagnetic sieve shaker (Bertel), using a 6-piece sieve for 20 minutes, to separate the sandy fractions. The fraction removed from each sieve was weighed and recorded, and the contents of sand, silt and clay were determined, according to the classification of [6].

#### 4.3.2 Organic matter

The quantity of Organic Matter (OM) was determined through the method of wet organic matter oxidation [4]. One gram of each lyophilized sediment was oxidized with 10 mL of K_2_Cr_2_O_7_ 1N within 20 mL of H_2_SO_4_ PA. After light agitation and resting for 30 min, 200 mL of distilled water, 10 mL of orthophosphoric acid PA and 1 mL of 1% diphenylamine were added. The excess of remaining dichromate from the oxidation was titrated with 0.5N ammoniacal ferrous sulfate (AFS) solution and the organic matter content was calculated based on the volume of AFS added in the titration. The same procedure was performed without sediment sample to obtain a blank value for comparison with the samples.

### 4.4 Chemical analysis

#### 4.4.1 Procedure 1 - inorganics in water

The concentrations of the inorganic chemicals in the water were determined by two different procedures. For the procedure 1, performed by the IO-USP Institution, the samples were filtered with PTFE membrane of 0.45 μm pore size (Merck Millipore^TM^), and analyzed in triplicate for determination of Al, As, Ca, Cd, Cr, Cu, Fe, K, Mn, Ni, Pb, Sc, Sr, Ti, V, Zn. The results were expressed as mg L^−1^ and compared to CONAMA Resolution 357/2005, according to its respective class of water (for Doce River, Class II). The quantification was performed in Inductively Coupled Plasma Mass Spectromer (ICP-MS) according to USEPA 6020 (2014).

For the procedure 2, performed by the UFPR Institution, the 48 river water samples were centrifuged for 10 minutes at 3500 rpm to precipitate the suspended material. Subsequently, the solutions were analyzed directly by conventional nebulization in ICP OES for elemental quantification of Al, As, Cd, Co, Cu, Fe, K, Li, Mg, Mn, Na, Ni, Pb, Ti, V, Zn. For quantification of Hg was used the hydride generation technique coupled to the ICP OES.

For instrumental determination it was used an Inductively Coupled Plasma Optical Emission Spectrometer (ICP OES, Thermo Scientific, model iCAP 6500) with axial view, software Thermo iTEVA - Control Centre version 2.8.0.97, K-TYPE Concentric Nebulizer and Cyclonic Spray chamber aqueous. PVC peristaltic pump tubing Orange/White, 0.64 mm i.d. (Meinhard Galss Products, USA) were used for the transport of the solutions. For hydride generation technique it was used a lab-made equipment with PVC peristaltic pump tubing White/White, 1.016 mm i.d. for transport of the sample solutions and PVC peristaltic pump tubing Black/Black, 0,76 mm i. d. for transport of the acid and reductant solutions. The instrumental parameters are shown in Table 10.

**Table 10:** ICP OES instrumental parameters for measuring inorganic contaminants in the water.

*Conventional Nebulization*		
Radiofrequency		40 MHz
Radiofrequency potential(RF)		1.15 kW
Plasma gas flow		12.0 L min^−1^
Auxiliary gas flow		0.5 L min^−1^
Carrier gas flow		0.7 L min^−1^
Stabilization time		20 s
Replicates		2
Torch		Quartz, axial view
Analytical lines	Al	396.152 nm
	As	193.696 nm
	Cd	228.802 nm
	Co	228.616 nm
	Cr	267.716 nm
	Cu	324.754 nm
	Fe	259.940 nm
	K	766.490 nm
	Li	670.784 nm
	Mg	279.553 nm
	Mn	257.995 nm
	Na	589.592 nm
	Ni	221.647 nm
	Pb	220.353 nm
	Ti	323.452 nm
	V	309.311 nm
	Zn	213.856 nm
*Hydride generation system*		
Sample flow		3.0 mL min^−1^
NaBH_4_ flow		3.0 mL min^−1^
HCl flow		3.0 mL min^−1^
NaBH_4_ concentration		1.0% m v^−1^ (0.4% m v^−1^ of NaOH)
HCl concentration		6.0 mol L^−1^
Analytical line	Hg	184.950 nm

The values of range concentration of analytical curves and the limits of quantification (LOQ) followed the values established by CONAMA Resolution 357/2005 (Doce River, Class II). The analytical curves were constructed from a 1000 mg L^−1^ multielement standard solution (AccuStar, New Have, USA) containing Al, Cd, Co, Cr, Cu, Fe, K, Li, Mg, Mn, Na, Ni, Pb, and Zn. For the other elements, standard single-element solutions of 1000 mg L^−1^and for Hg 100 mg L^−1^ were used. The concentration ranged from 1.0 to 2000 µg L^−1^ in 1% (v v^−1^) HNO_3_ concentrated medium for Al, As, Cd, Co, Cr, Cu, Fe, K, Li, Mg, Mn, Na, Ni, Pb and Zn. For determination of Hg by hydride generation, the concentration range was from 0.1 to 2.0 µg L^−1^ aqua regia (3HCl:1HNO_3_) 10% (v v^−1^). The curves showed a correlation coefficient above 0.999 for all the elements. The solutions for the generation of hydrides were prepared with NaBH_4_ P.A. (Reatec, Brazil), NaOH P.A. (Synth, Brazil), HCl P.A (Reatec, Brazil) previously distilled and HNO_3_ (Merck, Germany).

#### 4.4.2 Organic chemicals in water

The organic contaminants (Polycyclic aromatic hydrocarbons - PAHs) in the water were determined according to the method of Solid-Phase Extraction-SPE (Method 3535A) proposed by USEPA (2007), validated by [7] for the PAHs and according to [8] for the Pyrethroids . The compounds were extracted from filtered water samples (PTFE membrane of 0.45 μm pore size, Merck Millipore^TM^) through solid phase extraction with cartridge SPE C18 (Discovery Ò DSC-18 SPE, 0,5g/6 mL) coupled to a vacuum manifold system (Visiprep^TM^). The columns were preconditioned with Miliq water, in a flux of approximately 5 ml.min^−1^, and the compounds were eluted with 0.5 mL of acetone, 0.5 mL of ethyl acetate, 0.5 mL of hexane and lastly 0.5 mL of a mixture of the three solvents in equal proportions.

The determination of the organic contaminants was performed with a gas chromatography coupled to a mass spectrometer (GCMS-QP2010, Shimadzu) in the splitless mode. The analytes of interest were separated in a capillary column DB-5 Agilent J&W (5% diphenyl-95% dimethyl polysiloxane) of 30 m x 0.25 mm i.d with 0.25 µm film thickness, with helium (99.999%) as the carrier gas. The initial oven temperature was 60°C, that was maintained for 3 min at a rate of 15°C min ^-1^, after it was increased to 200°C at a rate of 6°C min ^-1^, until reaching the final temperature of 300°C that was maintained for 7 min. For identification of the 21 PAH compounds, the retention time of the analytical standards were used to match with the injected samples, considering the confirmations of the ion mass fragments and the NIST library. Quantification of analytes was performed based on a 5-point calibration curve, using the internal standard method. Accepted linearity was obtained in all calibrations (r^2^>0.99).

### 4.5 Sediment

#### 4.5.1 Inorganic chemicals

The trace elements Al, Cd, Cr, Cu, Fe, Mn, Ni, Pb, Sc, Ti, V and Zn were quantified in the whole sediments as triplicate samples for each of the eight sites and sampling season. All glassware was previously washed with 10% nitric acid. Metals were extracted from 1 g of sediment digested in 5 mL of nitric acid 50%, followed by two successive additions of 2.5 mL of HNO_3_ PA (Merck) in the digestive block at 95° C. Peroxide hydrogen (6 mL progressively added, for 270 min), HCl PA (5ml for 15 min) and water were added to complete 50 ml of solution. The detection was performed in an inductively coupled plasma mass spectrometry (ICP-MS). The metal concentrations in the sediment were expressed as μg.g^−1^ or dry (weighing after dehydration).

To ensure the reliability of the test, an SS-2 certified reference material (EnviroMAT Contaminated Soil, SCP Science) was subjected to the same analytical procedure. The certified values of each analyzed element are presented in Table 11, with recovery data and relative standard deviation. All recoveries were within the values recommended by USEPA (1996) showing accuracy and precision. Detection limits were calculated (m = 1.0 g; v = 50 ml) and with the exception of Cd (0.38 mg kg-¹) all results were above.

**Table 11:** Certified value (mg kg^−1^) and result of mean ± standard deviation (SD), recovery and relative standard deviation (RSD) of SS-2 (n=3) for inorganic chemicals measured by ICP IOS.

Al	9548	10125 ± 679	106	6.9
As	3.36	4 ± 0,5	115	12.2
Cd*	0.91	1.2 ± 0.1	130	5.2
Cr	92.6	102 ± 8	110	7.8
Cu	120	123 ± 5	102	4.3
Fe	23083	23118 ± 785	100	3.0
Ni	25.1	24 ± 1	96	5.0
Pb	244	219 ± 10	90	4.5
Zn	281	289 ± 22	106	7.7

#### 4.5.2 Total Hg in the sediments

The experimental procedure to determine total Hg in sediment samples was performed according to Santos et al. 2005. Sediment samples were firstly grounded in agate mortar and sieved with a 100 µm stainless steel granulometric sieve. In approximately 30 mg of the sieved sample it was added 1.5 mL of aqua regia (3HCl:1HNO_3_) in 15 mL polypropylene tubes. The mixture was submerged in an ultrasound bath for 30 minutes. After this period, the suspension was incubated for 24 hours with occasional manual agitation and was subjected to another ultrasound for 30 minutes. At the end the volume was adjusted to 15 mL with deionized water. The final concentration of aqua regia in the suspension was 10% v v^−1^. The procedure was performed in duplicate and analyzed according to the ICP OES conditions described for the Procedure 2.

Blank reagents were included in the analysis system to ensure the absence of contamination in all the samples. The recovery test was performed with the analysis of certified material RS-3 (river sediment) from a round-robin test, the recovery rate was 94% for Hg (Santos, *et al.* 2005).

#### 4.5.3 Organic chemicals in the sediments

Differently than inorganic analysis, sediments sampled for organic analysis were stored in aluminum containers as composite samples, formed by a mixture of three subsamples, in equal proportions (total of 8 composite samples per season).

The extraction of organic contaminants was carried out according to [9], consisting of the extraction of total organic contaminants followed by clean-up for separation into three fractions with different polarities. All glassware was previously washed with 25% Detertec and rinsed with acetone PA. For each 15 g of lyophilized sediment sample, it was spiked deuterated surrogate standards at final concentrations of 100 ng g^−1^(p-terphenyl- d_14_, purchased from Supelco/ Aldrich). The total organic chemicals from the spiked samples of sediment were extracted with a solid-liquid extraction consisting of the addition of a sequence of solvents with decreasing polarity indexes (*P`*): 25 ml of each solvent Acetone, Ethyl acetate, Dichloromethane, Hexane, and the mixture of the four solvents in same proportions, all purchased from MERCK. Samples remained on ultrasound for 20 min for each solvent, followed by centrifugation at 4000 rpm for 15 min. The supernatant was concentrated in a rotary evaporator in a 250 mL flask to a volume of approximately 1 ml.

The clean-up procedure consisted of separating the compounds through chromatographic affinity in an open preparative column (1 × 50 cm) containing 8.0 g of Silica gel (MERCK), 4.0 g of alumina (MERCK), 0.5 g of Cu (MERCK) and 1.0 g of anhydrous sodium sulfate (VETEC). The elution sequence was based on the increasing range of eluotropic strength by adding 40 ml of Hexane (Fraction 1), 60 ml of Hexane: Dichloromethane: Ethyl Acetate (3: 3: 1 - Fraction 2) and 50 ml of Dichloromethane: Methanol (9: 1 - Fraction 3). The extracts were concentrated in a rotary evaporator and stored in amber vials kept at -20° C until analysis. The determination of the 21 PAHs in the sediment extract (Fraction 2) (Table 8) and Pyrethroids (Fraction 3) (Table 9) was performed with a gas chromatography coupled to a mass spectrometer (GC-MS) as previously described for the water samples.

### 4.6 QA/QC

Method blanks were included in the analysis system to ensure the absence of contamination in all the samples. The method blank consists of clean glassware included in each batch under the same processing method than environmental samples. This quality control sample is used for sample concentration correction of environmental samples via background subtraction. Besides instrument blanks (n-hexane) were analyzed to evaluate any instrument noise.

Furthermore, the deuterated surrogate standard with known concentration, was spiked at in the beginning of the extraction and quantified at the end of all the process, to assess the efficiency of the analyses (Cavalcante et al., 2008. The average recovery from our environmental samples was 63% ± 29%. The values in the Table 8 were not modified by the recovery values.

## Ethics Statements

The authors confirm that a consent from RENOVA foundation was obtained for data collected from their website.

## CRediT Author Statement

**Yamamoto, F.Y.:** Conceptualization, Methodology, Validation, Formal analysis, Investigation; **Eufrásio, G.**^:^ Methodology, Validation, Investigation; **Nascimento, L.S.:** Methodology, Validation, Investigation, Writing – review & editing; **Fernandes, G.M.:** Methodology, Validation, Investigation, Writing – review & editing; **Santos, M.P.:** Methodology, Validation, Investigation, Writing – original draft preparation; **Kim, B.S.M.:** Validation, Investigation; **Figueira, R.C.L.:** Validation, Resources; **Cavalcante, R.M.:** Validation, Resources; **Grassi, M.T.:** Validation, Resources; **Abessa, D.M.S.:** Conceptualization, Resources, Writing – review & editing, Supervision.

## Declaration of Competing Interest

The authors declare that they have no known competing financial interests or personal relationships that could have appeared to influence the work reported in this paper.

## Data Availability

Chemical datasets of environmental samples (2019) from the Doce River Basin, Brazil (Original data) (Mendeley Data).
